# The Implications of Noncoding RNAs in the Evolution and Progression of Nonalcoholic Fatty Liver Disease (NAFLD)-Related HCC

**DOI:** 10.3390/ijms232012370

**Published:** 2022-10-15

**Authors:** Ioana Rusu, Radu Pirlog, Paul Chiroi, Andreea Nutu, Vlad Radu Puia, Alin Cornel Fetti, Daniel Radu Rusu, Ioana Berindan-Neagoe, Nadim Al Hajjar

**Affiliations:** 1 Department of Pathology, Regional Institute of Gastroenterology and Hepatology, 400162 Cluj-Napoca, Romania; 2 3rd Department of General Surgery, “Iuliu Hatieganu” University of Medicine and Pharmacy, 400186 Cluj-Napoca, Romania; 3 Research Center for Functional Genomics, Biomedicine and Translational Medicine, “Iuliu Hatieganu” University of Medicine and Pharmacy, 400337 Cluj-Napoca, Romania; 4 Department of Surgery, Regional Institute of Gastroenterology and Hepatology, 400162 Cluj-Napoca, Romania

**Keywords:** NAFLD, hepatocellular carcinoma, ncRNAs, miRNA, lncRNA

## Abstract

Nonalcoholic fatty liver disease (NAFLD) is the most prevalent liver pathology worldwide. Meanwhile, liver cancer represents the sixth most common malignancy, with hepatocellular carcinoma (HCC) as the primary, most prevalent subtype. Due to the rising incidence of metabolic disorders, NAFLD has become one of the main contributing factors to HCC development. However, although NAFLD might account for about a fourth of HCC cases, there is currently a significant gap in HCC surveillance protocols regarding noncirrhotic NAFLD patients, so the majority of NAFLD-related HCC cases were diagnosed in late stages when survival chances are minimal. However, in the past decade, the focus in cancer genomics has shifted towards the noncoding part of the genome, especially on the microRNAs (miRNAs) and long noncoding RNAs (lncRNAs), which have proved to be involved in the regulation of several malignant processes. This review aims to summarize the current knowledge regarding some of the main dysregulated, noncoding RNAs (ncRNAs) and their implications for NAFLD and HCC development. A central focus of the review is on miRNA and lncRNAs that can influence the progression of NAFLD towards HCC and how they can be used as potential screening tools and future therapeutic targets.

## 1. Introduction

Liver cancer is a frequent and aggressive cancer type, with 905,677 newly diagnosed cases and over 830,180 deaths worldwide in 2020 alone. The annual incidence of hepatic cancers is expected to exceed one million cases by 2025 [1,2]. Hepatocellular carcinoma (HCC) accounts for roughly 90% of all primary hepatic tumors, which makes it the sixth most common type of cancer worldwide and the third leading cause of cancer-associated mortality. Such a discrepancy between incidence and mortality emphasizes the aggressiveness and poor prognosis associated with this malignancy [3,4,5,6,7,8]. HCC prognosis is strongly linked with tumor stage, the best survival rates being reported in early diagnosed patients, whereas there is currently no curative option for advanced HCC stages [9,10].

NAFLD includes a heterogeneous variety of progressive conditions that range from steatosis (intrahepatic fat accumulation exceeding 5–10% by weight) to nonalcoholic steatohepatitis (NASH), which may progress for some patients to liver cirrhosis, HCC, and, ultimately, liver failure [11,12]. NAFLD remains the most common liver disease, with a worldwide prevalence of 25% [13,14]. This complex metabolic disorder has recently emerged as one of the main contributing factors to HCC development. The link between human cancer and viral infections has already been well documented [15,16]. However, although cirrhosis and chronic hepatitis B infection remain the main risk factors linked with HCC (in 50% of all cases), a growing amount of clinical evidence suggests that approximately 24% of the HCC cases are a consequence of neglected NAFLD [17,18,19,20,21,22]. This constant rise in NAFLD-related HCC relies on two distinct epidemiologic events. First, the growing incidence of other metabolic-syndrome-associated conditions, such as hyperlipidemia and excessive body weight, obesity, type 2 diabetes mellitus (T2DM), and insulin resistance [23,24,25], are jointly contributing towards hepatocarcinogenesis; thus, NAFLD is expected to become the leading cause of HCC [26,27,28,29,30,31,32,33,34] and the ultimate indication for HCC-related liver transplant candidates as well [35,36,37,38]. Second, with the successful implementation of anti-hepatitis B/C virus campaigns, the risk of viral-driven HCC has significantly decreased, NAFLD emerging as a primary risk factor for HCC [39]. Altogether, these two epidemiological events are promoting NAFLD as one of the primary causes of HCC.

Hepatocarcinogenesis can occur in both cirrhotic and noncirrhotic livers. However, since the presence of a pre-existing cirrhotic state has been reported in up to 80–90% of HCCs, screening is currently recommended for cirrhotic patients only, which leaves uncovered a significant part of early-stage HCC cases, especially since noncirrhotic patients are at elevated risk compared to the general population being frequently clinically silent. Therefore, noncirrhotic, nonviral, NAFLD-related HCC cases are usually diagnosed late, with limited therapeutic options. This emphasizes the need for a clearer understanding of the mechanisms underlying the steatosis–hepatocarcinogenesis transition, which could aid in discovering novel genetic biomarkers and in developing better screening methods [40,41,42,43].

Previous studies on liver tumorigenesis focused on exploring the genome’s protein-coding regions due to their central role in protein synthesis. However, less than 2% of the human genome encodes proteins, whereas, in the remaining 98% of the DNA, noncoding sequences and their RNA transcripts, generally known as noncoding RNAs (ncRNAs) can be found. These transcripts are valuable regulators of gene expression and are involved in modulating different biological processes [44,45]. Thus, increasing evidence indicates that multiple evolutionarily conserved ncRNAs, such as microRNA (miRNA) and long noncoding RNA (lncRNA), are highly involved in different molecular processes, including those associated with the pathogenic transition of steatosis to hepatocarcinoma [46,47].

In recent decades, ncRNAs have become one of the central focuses in cancer research. MiRNAs are endogenous, 19–24 nucleotides long, single-stranded RNA molecules that can modulate, at the post-transcriptional level, different complementary target messenger RNAs involved in many pathophysiological processes, including those associated with steatosis and hepatocarcinogenesis [48,49,50]. From the same group of transcripts but distinct through their structure and functions, lncRNAs are a highly conserved subgroup of ncRNAs, exceeding 200 nucleotides in length, with limited known protein-coding potential, that have recently emerged as significant contributors in the pathophysiology of various human conditions, entangling chronic liver diseases, such as NAFLD and HCC [51,52].

As these ncRNAs are differentially expressed in dependence on the hepatic state of the organism, exploring the miRNAs and lncRNAs’ involvement in the NAFLD-related HCC development is currently of primary interest, yet not a fully understood topic, so extensive research will provide a better view on the molecular mechanism behind the steatosis–hepatocarcinogenesis transition [53,54]. In this regard, many researchers are now conducting intensive studies designed to identify novel and reliable biomarkers that could be further validated and translated into screening panels [55,56]. This review aims to provide an updated view on the critical roles of miRNAs and lncRNAs in the pathologic transition of steatosis to hepatocarcinogenesis, highlighting the potential use of such ncRNAs as diagnostic biomarkers and possible future therapeutic targets for NAFLD-related HCC.

## 2. Current Epidemiologic Status of NAFLD-Related HCC

Within the NAFLD spectrum, NASH is the main risk factor for HCC development, as it can lead to advanced fibrosis and cirrhosis [38,57]. The cumulative annual incidence for developing NASH-related HCC is about 2.4–12.8% [58]. However, in the absence of NASH or cirrhosis, NAFLD remains the main underlying condition for hepatocarcinoma, which is usually less likely to be diagnosed by surveillance compared to HCC that develops in the setting of NASH, cirrhosis, or viral hepatitis [59,60]. Furthermore, implementing anti-hepatitis B/C virus campaigns decreased the risk of virally driven HCC. Along with the global rise in obesity and T2DM, a constant increase in NAFLD-related HCC incidence has been noted. Thus, NAFLD has rapidly emerged as a primary risk factor for HCC [39].

One quarter of the global population currently suffers from NAFLD [61,62]. This silent liver condition is the fastest growing cause of HCC in several regions worldwide, including the USA, China, Germany, France, and the UK [63], while being closely and bidirectionally connected with different metabolic-syndrome-associated conditions [64,65]. NAFLD is an underlying disorder in up to 68% of T2DM patients and between 60 and 95% of obese individuals [66,67].

In the past seven decades, a comprehensive, systematic review study by Orci et al. focused on assessing the incidence of HCC in patients with NAFLD. The results indicated, with a 95% confidence interval, that the noncirrhotic NAFLD-related HCC rate was 0.03 per 100 person-years, while the rate of cirrhotic NAFLD-related HCC was higher, reaching 3.78 per 100 person-years. Moreover, in cirrhotic patients undergoing regular HCC screening, the incidence rate was higher, at 4.62 per 100 person-years [68]. In another literature study, Pinyopornpanish et al. found that, out of all the 1110 NAFLD-related HCCs included in their research, 15% occurred in noncirrhotic patients [40], highlighting the importance of HCC screening in all NAFLD individuals, regardless of the cirrhotic status. Overall, the incidence of HCC has increased throughout the past several decades. Although viral HBV/HCV infections and cirrhosis appear to remain the most significant risk factors, the connection with metabolic-syndrome-related disorders, the clinically silent symptoms, and the rapidly growing incidence of NAFLD make this liver etiology one of the important causes of HCC development [5,69,70,71].

However, the current clinical guidelines do not include abdominal ultrasound screening for noncirrhotic patients. However, about half of the NAFLD-related HCC cases occur without an established cirrhosis [41,60,72,73]. This unmet need in the screening and surveillance of NAFLD-related HCC patients is a clinical management disruption that could be prevented. Nonetheless, despite all the advances in surgical procedures and chemotherapeutic formulations, NAFLD-related HCCs have a lower receipt of curative therapy with poorer survival rates. Therefore, although several targeted therapies have been previously approved [74] or investigated in different human cancers [75], due to its high prevalence, suboptimal screening and surveillance clinical guidelines, poor prognosis, and limited treatment response, NAFLD-related HCC remains a major global health problem [76,77].

## 3. Understanding the Pathogenesis of NAFLD-Related HCC

Histologically, NAFLD encompasses a broad spectrum of progressive clinicopathological states that include steatosis, a benign form of nonalcoholic fatty liver (NAFL), followed by a necroinflammation subtype called nonalcoholic steatohepatitis (NASH), which is characterized by hepatic inflammation, the presence of hepatocellular injury (hepatocyte ballooning), and fibrosis, which could further progress to liver cirrhosis, HCC, and eventually liver failure [39,56,61,78]. Hepatocarcinogenesis triggered on a lipotoxic, chronically inflamed liver has an elevated incidence in patients without cirrhosis, as up to 50% of NAFLD-related HCCs occur in noncirrhotic conditions [38,79]. Several research articles reported that patients with NAFLD-related HCC usually have a worse prognosis when compared with HCC developed based on other liver etiologies. This is due to corroboration of different factors, including patients usually being diagnosed at old age when they are already experiencing various other comorbidities and the lack of screening surveillance in noncirrhotic NAFLD, which leads to late-stage diagnosis when therapeutic options are limited [80,81,82,83,84,85].

The development of NAFLD-related HCC is a complex, multistep, multifactorial, and not yet fully understood process. NAFLD is described as a hepatic metabolic disease triggered by excessive fat accumulation in the liver due to a fat-rich diet, disrupted metabolic pathways, and genetic and epigenetic factors (ncRNAs and DNA methylation) [86,87]. Various pathophysiological alterations, such as insulin resistance, precise cytokine release, oxidative stress, and mitochondrial damage, are involved in the transition of NAFLD to HCC. In the background of a pre-existent NAFLD, obesity and lipotoxicity-mediated insulin resistance are significant contributors to HCC pathogenesis through systemic inflammation and the promotion of oncogenic pathways [88]. Moreover, the increased hepatic lipid storage, especially of free fatty acids (FFA) and diglycerol, can facilitate endoplasmic reticulum (ER) stress and reactive oxygen-species-mediated DNA damage, which leads to a chronically inflamed hepatic environment, resulting in NASH and liver fibrosis, which will further drive the oncogenesis of NAFLD-related HCC [86,89].

It is well established that IL-6, JAK, and STAT signaling pathways are essential drivers in the development and progression of the HCC [90,91,92]. In a recent study by Grohmann et al., it was shown that, in a hepatic oxidative environment in the context of obesity, STAT-1 and STAT-3 signaling are enhanced via the inactivation of STAT-1 and STAT-3 phosphatase T cell protein tyrosine phosphate. They also showed that STAT-1 signaling leads to T cell recruitment and the development of both NASH and fibrosis but not HCC. In contrast, STAT-3 signaling was responsible for NAFLD-related HCC development independent of NASH and fibrosis [93]. Finally, genetic factors are also involved in the pathogenesis of NAFLD-related HCC. Primarily, the genetic variants identified so far, PNPLA3, TM6SF2, and MBOAT7, increase the severity of NAFLD via elevating hepatic lipid storage levels by hydrolyzing triglycerides and retinyl esters, hampering lipid mobilization, and decreasing lipoprotein export, thus supporting the progression and development of HCC [94,95,96,97,98].

## 4. The Implications of ncRNAs in the Pathogenesis of NAFLD-Related HCC

The advances made in genome sequencing achieved over the past decades revealed that potentially between 97 and 98% of the human genome is transcribed into ncRNAs, which do not code for proteins but are somewhat involved in DNA replication, RNA splicing, translation, and epigenetic regulation of various biological processes, including hepatocarcinogenesis [99]. Based on their sequence length, these noncoding transcripts are divided into two groups: short ncRNAs containing less than ~200 nucleotides and long noncoding RNAs exceeding this threshold. In recent years, the expression of ncRNAs, especially miRNAs and lncRNAs, has been extensively studied and linked with several pathological changes, including the transition of steatosis to hepatocarcinoma [100,101,102].

Since their discovery, miRNAs have augmented the research on NAFLD and HCC pathogenesis, especially as they proved to be involved in the molecular events underlying the transition between the two. In recent years, alteration of specific hepatic miRNAs has been linked with several key hallmarks of both NAFLD and HCC. In contrast, few studies have investigated their implication in NAFLD-related HCC progression. Hence, specific hepatic miRNAs have been shown to regulate lipid metabolism, glucose homeostasis, cell proliferation, apoptosis, migration, and differentiation in NAFLD, HCC, or NAFLD-related HCC, thus being considered novel potential molecular biomarkers associated with these liver diseases [103,104,105,106,107].

### 4.1. miRNA Biogenesis

The biogenesis of these miRNAs is a multistep process that starts in the nuclei of hepatic cells [108,109]. Firstly, RNA polymerase II transcribes primary miRNAs (pri-miRNAs) transcripts within the canonical/mirtron pathway. Thus, the 5′ ends of pri-miRNAs are capped, while the 3′ ends are polyadenylated [110]. Then, a nuclear Rnase III enzyme named Drosha cleaves the pri-miRNAs into 70-100-nucleotide hairpin-structured precursors called pre-miRNAs [111]. Next, pre-miRNAs are exported into the cytoplasm, where they bind with exportin-5 and Ran-GTP to be further cleaved by an endoribonuclease, known as Dicer, into double-stranded pre-miRNAs [112,113]. Subsequently, these newly formed double-stranded pre-miRNAs undergo a fast unwinding by loading onto the AGO protein. Only one strand serves as a guide to target mRNAs, remaining bounded. Finally, the RNA-induced silencing complexes (RISC) and the AGO proteins form the mature miRNAs [50,114]. These mature noncoding transcripts, of which expression is often dysregulated, are ready to modulate the mRNA degradation and translational inhibition, regulating several targets involved in the pathogenesis of NAFLD-related HCC [115].

### 4.2. LncRNAs

**Figure 1:**
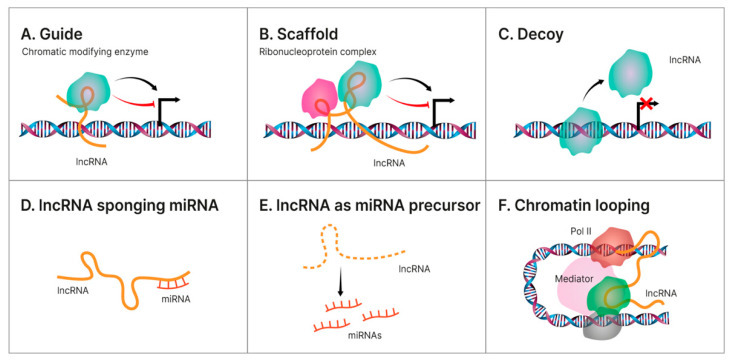
Different mechanisms of action for lncRNAs. Illustration of the main regulatory mechanism endorsed by hepatic lncRNAs in the pathology transition of NAFLD towards HCC. (**A**). Guide—some lncRNAs aid specific proteins to reach their target, thus accomplishing their biological functions. Oftentimes, some of these proteins are transcription factors located on specific DNA regions and this guidance becomes an indirect way of regulating gene transcription; (**B**). Scaffold—LncRNA can facilitate the interaction of numerous molecules and proteins, enabling the assembly of different macromolecular complexes, thus promoting the conjunction and integration of molecular information within different signaling pathways; (**C**). Decoy—some lncRNAs bind directly to some protein molecules impairing their functions; (**D**). lncRNA sponging miRNA—some lncRNAs can act as ceRNAs, or sponges, reducing their effect upon the target mRNA, thus regulating gene expression; (**E**). lncRNA as miRNA precursor—some lncRNAs can act as precursors of miRNAs to directly modulate their regulatory activity; (**F**). Chromatin looping—as lncRNAs are long and mobile, they could serve as bridges to drive the inter- or intra-chromosomal interactions.

LncRNAs represent a large and heterogeneous group of >200 nucleotide-length transcripts that lack an open reading frame, are frequently poorly expressed in comparison with the levels of protein-coding genes, and are less conserved than mRNAs having a high cell/tissue-specific expression [116,117,118]. Currently, the involvement of lncRNAs in the pathogenesis of NAFLD-related HCC is an evolving subject with promising results [51,119]. In terms of functionality, there are currently four molecular patterns describing the mechanism behind the regulatory activity of lncRNAs in NAFLD-related HCC. Hence, lncRNAs can act as: (a) a molecular guide that binds to the target directing their localization; (b) a molecular scaffold by mediating the protein–RNA interactions; (c) a molecular decoy that directly binds to the targeted proteins/miRNAs suppressing their functions; (d) a miRNA sponge; (e) a miRNA precursor; and as (f) a molecular signal regulating gene expression by interacting with transcription factors or chromatin-modifying enzymes [120,121,122,123]. Nonetheless, and often in direct relation to different miRNAs, lncRNAs are currently investigated for playing a regulatory role in the multistep process of steatosis–hepatocarcinoma progression [46,52]. A visual representation of the lncRNAs mechanisms of action is illustrated below in Figure 1.

Recently, many findings emerged supporting the implication of miRNAs and lncRNAs in the pathogenesis of NAFLD-related HCC [124,125]. Dysregulated expression profiles of such ncRNAs could improve our understanding of this complex disease and hopefully provide the means for the development of noninvasive and cost-effective methods of diagnosis. In addition, most miRNAs and lncRNAs have either tumor-promoting or suppressive roles that can be exploited as therapeutic targets. 

## 5. Dysregulated miRNAs and Their Role in NAFLD-Related HCC

**Figure 2:**
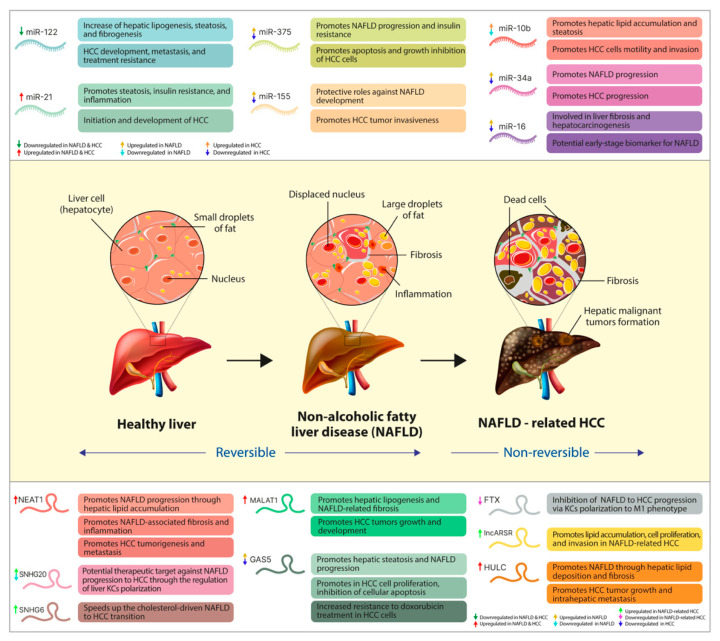
The pathologic transition of healthy liver towards NAFLD-related HCC, together with the main dysregulated ncRNAs and the disease-promoting processes they regulate. In the upper part of the figure, we illustrated the primary miRNAs and their expression pattern throughout the transition of NAFLD towards HCC, together with the pathological processes of which regulation they play a part in. Centrally, we summarize the main changes in the cellular and tissue architecture of the liver in both NAFLD (reversible) and NAFLD-related HCC (nonreversible). At the bottom, we presented the main lncRNAs involved in NAFLD transition towards HCC, their dysregulated expression level, and the main disease-promoting process in which they play a regulatory role.

NAFLD is associated with a dysregulation of hepatic metabolism, and, in the past decade, ncRNAs have been positioned among the primary regulators of these metabolic alterations [126]. The epigenetic mechanisms underlying several deregulations in the expression and function of specific miRNAs were involved in NAFLD development and its progression towards HCC [127]. Thus, several miRNAs were reported as critical regulators of the insulin signaling pathway, carbohydrate metabolism and hepatic glucose output, cholesterol and fatty acid metabolism (pathological accumulation of cholesterol and fatty acids), ER stress and autophagy, and the proinflammatory responses [50,128]. The cellular and tissue changes that occur in the hepatic architecture throughout the pathologic transition of healthy liver to NAFLD and further towards HCC [46,129], together with the main dysregulated miRNAs that modulate these disease-promoting processes, are illustrated in Figure 2.

Chronic liver inflammation underlying different hepatic etiologies, including NAFLD, is associated with reduced miR-122 expression in hepatocytes [130,131]. The relatively low levels of miR-122 expression remained consistent in NAFLD-HCC compared to healthy tissues [132,133]. Several studies link this miR-122 downregulation with an aggressive HCC phenotype. For example, in vitro analysis of Coulouarn et al. on HCC-derived cells (PLC/PRF/5, Huh-1, and Hep40 exhibited very high levels of miR-122, whereas Hep3B and HepG2 expressed little miR-122, and SNU387 and SNU398 did not express it at all) and on primary liver tumors and 28 matched nontumor surrounding liver tissues concluded that loss of miR-122 expression coincided with the acquisition of an invasive phenotype and suppression of hepatic functions, while being associated with a poor prognosis [134]. Independently, Tsai et al. found that liver-specific miR-122 is significantly downregulated in metastatic Mahlavu and SK-HEP-1 HCC cells and negatively regulates tumorigenesis. It appears that ADAM17, a direct target of miR-122, plays a role in HCC metastatic progression. Silencing of ADAM17 resulted in an apparent reduction in in vitro migration, invasion, tumorigenesis, and angiogenesis in the hepatic models of nude mice, similar to the restoration of miR-122 expression [135].

Kojima et al. studied the correlation between miR-122 expression level and α-fetoprotein (AFP), a widely used biomarker in HCC surveillance. They demonstrated that the miR-122/CUX1/miR-214/ZBTB20 pathway regulates AFP expression, and the miR-122/CUX1/RhoA pathway regulates the aggressive characteristics of such hepatic malignancy [136]. Wu et al. reported that miR-122-knockout mice display increased lipogenesis, changes in lipid secretion, IL-6 and TNF-α production, and upregulation of chemokine ligand 2, thus rapidly developing NASH. They also noted that miR-122 expression enhances fibrogenesis by inducing HIF1α and MAPK1, facilitating HCC development [137]. An extensive and comprehensive study by Akuta et al. investigated the impact of serum miR-122 on the histological features of HCC in a 305 NAFLD patient cohort. The research group saw a clear bidirectional relationship between serum levels of miR-122 and the severity of steatosis, ballooning, lobular inflammation, and disease stage of NAFLD-related HCC. In fact, during the longitudinal evaluation, the scientists observed a decreased tendency in the serum levels of miR-122, even before the fibrotic stage [138]. Tsai et al. found that miR-122-deficient mice exhibit inflammation and extensive lipid accumulation (steatosis), highlighting the anti-inflammatory role of this abundant hepatic miRNA [139]. The aberrant expression of many other miRNAs, including the hepatic abundant miR-29, miR-155, and miR-21 have been found associated with HCC-related chronic inflammation [140]. Overall, hepatic miR-122 deregulation appears to be involved in NAFLD-HCC progression, with its circulating levels correlated with disease stages. However, although several attempts were already made to integrate this miRNA into various HCC diagnostic panels, further validation of these miRNAs is still required as a valid biomarker [141].

The clinical utility of miR-21 in human cancer has been well studied [142]. Hepatic miR-21 expression is elevated in patients and animal models with confirmed NAFLD [130,143,144] and livers of HCC individuals [145,146]. Still, its circulating levels are inconsistent in serum or plasma samples [147,148]. MiR-21 was found upregulated in many hepatic-associated processes of NAFLD patients, including physiological ones, such as inflammation, fibrosis, and carcinogenesis, being a potent promoter of HCC [149]. In NAFLD, miR-21 modulates glucose and lipid metabolism in hepatocytes through a complex transcription network. In a study conducted in 2016, Calo et al. reported that miR-21, an oncomiRNA overexpressed in HCC and other liver etiologies characterized by the presence of steatosis, promotes hepatic insulin resistance and lipogenesis in obesogenic-diet-fed mice through a fine regulation of Foxo1, Insig2, STAT3, and HNF4-α. The research group concluded that hepatic miR-21/miR-21* deficiency is a viable approach for preventing glucose intolerance and steatosis in mice, thus revealing the potential of miR-21/miR-21* as a therapeutic target for NAFLD [150]. The same year, Wu et al. confirmed the presence of elevated miR-21 levels in high-fat diet-fed mice, where it promoted hepatic lipid accumulation and cancer progression by interacting with the Hbp1-p53-Srebp1c pathway, thus endorsing miR-21 as a link between NAFLD and HCC but also as a potential therapeutic target for both disorders [151]. Su et al. provided experimental data supporting the involvement of miR-21 in regulating triglyceride and cholesterol metabolism in an in vitro model of NAFLD HepG2 cells via the inhibition of HMGCR expression [147]. Loyer et al. revealed that, through the inhibition of the PPARα signaling pathway, hepatic miR-21 contributes to hepatocyte injury, inflammation, and fibrosis [143]. These results indicate that miR-21 could be a viable biomarker for diagnosing and treating NAFLD.

Several studies confirmed that miR-21 induces hepatic fibrosis by simultaneously activating hepatic stellate cells (HSCs) via PTEN/Akt signaling [152], hepatocyte EMT by targeting SPRY2 or HNF4α [153], oxidation, and collagen synthesis through the enhanced activity of AngII upon the NLRP3 inflammasome via Spry1/ERK/NF-κB and Smad7/Smad2/3/NOX4 pathways [154,155]. As expected, several studies showed that loss of miR-21 expression results in decreased steatosis, inflammation, collagen storage, and impairment of fibrosis [156,157]. In patients with HCC, miR-21 can promote migration, invasion, and progression through the miR-21-PDCD4-AP-1 feedback loop [158] and the activation of PTEN, which further triggers AKT by interacting with phosphatidylinositol 3-kinase signaling pathway [145]. In addition, Wang et al. reported that overexpressed hepatic miR-21 is responsible for the inhibition of KLF5 w, which leads to HCC migration and invasion [158]. At the same time, Zhou et al. showed that exosomal miR-21 directly targeted PTEN, activating PDK1/AKT signaling in HSCs and promoting HCC progression by secreting a complex repertoire of angiogenic cytokines, including VEGF, MMP2, MMP9, bFGF, and TGF-β [159]. Finally, Wagenaar et al. demonstrated that treatment with specific single-stranded oligonucleotide inhibitors of miR-21 led to suppression of HCC growth in two separate xenograft models, highlighting the role of miR-21 in the maintenance of a tumorigenic phenotype in HCC [146]. Furthermore, multiple clinical data confirm the overexpression of miR-21 in tissue and serum samples, noticing a significant correlation between this oncomiRNA and tumor progression, thus suggesting its potential role as a biomarker for HCC prognosis [160,161,162,163]. Taken together, overexpression of miR-21 was confirmed in both NAFLD and HCC patients, with an apparent involvement in their pathogenesis, thus considered a potential link between the two diseases. [151,164].

Another highly conserved transcript, miR-375, was overexpressed in serum samples of NAFLD-confirmed patients compared with healthy individuals and was significantly associated with disease severity, thus being considered a potential biomarker for NAFLD progression [165]. In 2018, Lei et al. confirmed the genuine overexpression of miR-375 in serum samples of HFD-fed mice and correlated it with the decreased expression of AdipoR2, a direct target of miR-375. In this regard, the researchers noted that inhibition of miR-375 in human HCC cells HepG2 caused an apparent upregulation in adiponectin expression, while downregulating leptin, TNF-α, and IL-6 levels. Hence, miR-375 is involved in NAFLD progression and was proposed as a novel therapeutic target [166].

Regarding the expression and roles in HCC pathogenesis, Li et al. showed that miR-375 is significantly downregulated in human HCC tissues and cell lines (Huh-7, SK-HEP-1, MHCC97-H, MHCC97-L, and Hep3B2.1–7). At the same time, its induction resulted in the in vitro promotion of apoptosis and the inhibition of HCC proliferation. Furthermore, the scientists identified that ErbB2, a member of the epidermal growth factor receptors, is a direct target of miR-375 and is believed to play an essential role in the development and progression of HCC. Induction of miR-375 decreased the expression of ErbB2, while downregulation of ErbB2 was found to inhibit the growth of the HCC cell [167]. In a similar study, He et al. confirmed the significant downregulation of miR-375 in HCC tissues (60 pairs of HCC and matched adjacent nontumor tissues) and cell lines (SK-HEP-1, HepG2, Hep3B, Huh-7, MHCC97-H, and MHCC97-L compared with normal hepatocytes). By Liu et al., the induced overexpression of miR-375 was associated with an in vitro decrease in hepatocyte proliferation, clonogenicity, and invasion, and also with induced G1 arrest and apoptosis. AEG-1, which is a downstream regulator of oncogenic Ha-Ras and c-Myc, was found to be negatively regulated by miR-375. Thus, downregulation of miR-375 leads to AEG-1 overexpression, which activates the PI3K/Akt, NF-κB, and Wnt/β-catenin signaling pathways, reversing the antitumor effects of miR-375 deprivation in HCC [168,169]. Another direct and negatively regulated target of miR-375 is YAP, an oncogene involved in the HCC development [169]. Therefore, miR-375 may represent an important molecular link between NAFLD-associated insulin resistance and hepatocarcinogenesis, playing a part in the pathogenesis of both conditions.

Zheng et al. investigated a different mechanism for the pathogenesis of NAFLD and HCC through miR-10b, another ncRNA involved in the hepatic lipid metabolism and an active contributor to liver steatosis by modulating PPAR-α expression [170]. Celikbilek et al. reported the overexpression of miR-10b in steatosis hepatocytes while promoting intracellular lipids and triglyceride accumulation. However, serum levels of miR-10b were found to decrease in NAFLD patients and were inversely associated with the degree of liver inflammation [171]. In 2014, Liao et al. found the expression levels of miR-10b to be increased in HCC tissues and cell lines (MHCC-97L), which promoted HCC cell motility and invasion through the regulation of HOXD10/RhoC/uPAR/MMPs axis [172]. Jiang et al. noticed that serum levels of miR-10b progressively increased throughout the transition from the healthy liver to chronic hepatic disease and towards HCC. This progressive increase in miR-10b expression across the NAFLD-HCC change could imply its use in the surveillance of NAFLD patients in the HCC [173].

Another miRNA thought to play a part in the NAFLD-HCC progression is miR-34a, which was investigated by Castro et al. and found to be upregulated in NAFLD patients where it reflected the severity of the associated steatohepatitis. In addition, the researchers noted an inverse correlation between SIRT1 and disease severity, and a positive one between p53 acetylation and NAFLD progression, representing direct targets of miR-34a [174]. The expression level of miR-34a was also upregulated in serum samples of NAFLD-confirmed patients [175]. However, regarding HCC, miR-34a was downregulated in liver tissues of HCC patients and associated with tumor invasiveness and metastasis [176]. In a recent study by Sun et al., the lower expression level of miR-34a in comparison with nontumoral tissues was confirmed. Furthermore, miR-34a expression was negatively associated with the regulation of HDAC1, one of its direct targets, which is generally found to be overexpressed in HCC tissues and correlated with cancer-specific mortality [177,178,179]. Hence, miR-34a was involved in the pathogenesis of both NAFLD and HCC; additional investigation is required to understand the mechanisms that make miR-34a a link between these two hepatic diseases.

Research on murine models conducted by Miller et al. showed that miR-155 expression is elevated and exhibits protective effects upon the development of NAFLD. In part, these findings were correlated with the interaction between hepatic miR-155 and its target LXRα, which prevented the excessive lipid accumulation in the liver of murine NAFLD mice [180]. Lin et al. further confirmed the protective roles of miR-155 in a study conducted on liver samples from transgenic mice where the hepatic overexpression of miR-155 was found to alleviate NASH development through one of its direct targets Ces3/TGH [181]. In the context of steatosis to hepatocarcinogenesis progression, miR-155 was significantly upregulated in the early stages of hepatocarcinogenesis, alongside the reduced expression of its target C/EBPβ, a tumor suppressor frequently inhibited in HCC. In addition, the research group reported an association between the ectopic expression of miR-155 and the growth of HCC cells, while its downregulation inhibited their growth [182]. The oncogenic behavior of hepatic miR-155 was also confirmed by Yan et al. They correlated its elevation with the downregulation of SOCS1, which activated STAT3, previously confirmed as an important prognostic biomarker in other cancers, such as glioblastoma [183], and further downregulated MMP9 expression, consequently promoting HCC tumor invasiveness [184]. Research on animal models shows that hepatic miR-155 overexpression has a protective role in NAFLD and, paradoxically, a tumor-promoting one in HCC [185]. Therefore, although extensive data are required to understand its implication in NAFLD-HCC progression, miR-155 was found to be preferentially accumulated in the liver after the administration of exosomes containing synthetic miR-155 mimics into miR-155-knockout mice. This preferential distribution of miR-155 in the liver shows its potential as a disease modulator in both conditions [186].

Multiple studies reported increased circulating levels of miR-16, a transcript known for its involvement in liver fibrosis and hepatocarcinogenesis, in patients with histologically confirmed NAFLD [187,188,189]. However, it appears that plasma levels of miR-16 decrease with NAFLD progression, down to the point of being downregulated in HCC, mainly in patients with tumors over 5 cm in diameter [190]. Thus, lower tissue and serum levels of miR-16 were confirmed by several studies conducted on HCC patients and further proposed as reliable diagnostic biomarkers. The miR-16 level transition can be used as a monitoring biomarker to assess the NAFLD progression sequentially. A biomarker panel composed of miR-16 with AFP, AFP-L3%, and DCP was reported to offer more sensitivity and specificity for HCC patients with smaller tumors [191,192]. Taken together, alone, or in combination with other biomarkers, miR-16 could be a valuable biomarker. Still, extensive research is required to understand better its roles behind the transition from simple steatosis to hepatocarcinogenesis. A summary of other dysregulated miRNAs that could play a part in the NAFLD-related HCC pathogenesis is presented in the following table (Table 1).

## 6. Dysregulated lncRNAs and Their Role in NAFLD-Related HCC

The other primary class of ncRNAs that has recently emerged as a significant contributor in the NAFLD-related HCC progression is the lncRNAs [51,119]. In recent years, several research studies explored the roles of lncRNAs in the pathogenesis of NAFLD and HCC and in some of the molecular events that occur during the transition between these two chronic hepatic conditions [54,214]. The cellular and tissue changes that occur in the hepatic architecture throughout the pathologic transition of healthy liver to NAFLD and further towards HCC [46,129], together with the main dysregulated lncRNAs that modulate these disease-promoting processes are illustrated in Figure 2.

In 2017, Wang et al. used an in vivo HFD-induced NAFLD SD rat model and an in vitro FFA-exposed BRL3A rat cell line to study the involvement of NEAT1 in the development and progression of NAFLD. Their findings showed increased levels of NEAT1 and mTOR signaling-pathway-associated protein, consistent in both in vivo and in vitro models of NAFLD. To assess the effects of NEAT1, the researchers transfected the rat hepatic BRL3A cells with either pcDNA-NEAT1 lentivirus or si-NEAT1. Overexpression of NEAT1 was positively correlated with the upregulation of acetyl-CoA carboxylase (ACC) and fatty acid synthase (FAS), both of which take part in NAFLD pathogenesis. On the other hand, the FFA-exposed BRL3A cells transfected with si-NEAT1 presented a reversed phenotype. Similarly, overexpression of NEAT1 increased the levels of p-mTOR and p-p70S6K1, while inhibition of NEAT1 showed the contrary effect. Finally, after a 4-week treatment with si-NEAT1 lentivirus, the triglyceride and cholesterol levels were alleviated in the animal model. These findings support the involvement of lnc-NEAT1 in NAFLD through regulating the h mTOR/S6K1 signaling pathway in rats. At the same time, its downregulation is proposed as a potential treatment for NAFLD [215].

In 2019, the upregulation of NEAT1 in hepatic models of NAFLD was confirmed by two separate studies and implicated in the miRNA-based mechanisms involved in NAFLD development. Sun et al. found that upregulation of NEAT1 was positively associated with the expression levels of miR-140 in liver tissues of C57 NAFLD mice and FFA-treated HepG2 cells, both responsible for NAFLD progression by inactivating AMPK/SREBP-1 signaling pathway. Silencing of miR-140 inhibited NEAT1 expression, decreasing the lipid deposition in HepG2 cells [216]. The same year, Chen et al. found that upregulation of NEAT1 inactivates the AMPK/SREBP-1 signaling pathway by sponging miR-146a, thus liberating the expression of ROCK1, promoting NAFLD progression through hepatic lipid accumulation [217]. Nonetheless, NEAT1 was involved in regulating NAFLD-associated fibrosis and inflammation, besides regulating lipid metabolism. Jin et al. reported that knockdown of NEAT1 inhibited GLI3 and promoted miR-506 expression, while overexpression of miR-506 silenced both NEAT1 and GLI3 in an in vitro model (BRL3A rat cell line). Thus, the authors concluded that NEAT1 plays a critical role in hepatic fibrosis and inflammation by regulating the miR-506/GLI3 axis [218]. Regarding its involvement in HCC development and progression, lnc-NEAT1 has been extensively studied. In 2015, Guo et al. conducted one of the first such projects. They reported an elevated expression of NEAT1 in the liver tissue samples from 95 HCC patients, which was further associated with several hallmarks of HCC, including the number of tumor nodes, metastasis, clinical TNM stage, the status of portal vein tumor embolus, vascular invasion, and the infiltration of tumor cells. Thus, authors consider NEAT1 as a pivotal player in tumorigenesis and metastasis of HCC [219]. In 2017, Fang et al. reported that upregulated NEAT1 promotes HCC progression by regulating a miR-129-5p-VCP-IκB [220]. In 2020, Zhang et al. came to a similar conclusion, as NEAT1 expression was found to be significantly upregulated in Huh-7 and MHCC-97H HCC cell lines compared with the HSC LX-2 cells. MiR-230a, a direct target of NEAT1, was significantly downregulated in HCC cells, confirmed in an in vivo nude mouse model. Moreover, a dual-luciferase activity assay identified LAGE3, a prognostic biomarker associated with progression, as a direct target of miR-320a. The researchers concluded that NEAT1/miR-320a/LAGE3 axis participates in HCC development and that NEAT1 could be a potential therapeutic target [221].

In addition, the researchers observed that induced overexpression of NEAT1 abolished the inhibitory effects of miR-504 on HCC cell viability, migration, and invasion, thus promoting cellular apoptosis. The underlying mechanism proved to be the negative regulation of miR-504 on SMO expression levels. Hence, the NEAT1/miR-504/SMO axis deserves a closer look, especially since the downregulation of NEAT1 alleviated HCC hallmarks in vitro, which promotes NEAT1 as a valuable molecular target for HCC treatments [222]. Yeermaike et al. observed that m6A demethylase ALKBH5 is responsible for the upregulation of NEAT1 in human HCC cell lines SMMC-7721, Huh-7, and L02, which further regulates HCC cell proliferation and migration by sponging miR-214 [223]. Based on these studies, the relationship between NEAT1 and miRNAs appears to be involved at different levels in HCC pathogenesis. Therefore, it deserves a more in-depth analysis to better understand and benefit from these findings. Moreover, Sakaguchi et al. continued the in vitro studies on human HCC cell lines, Huh-7, HLF, and Huh-6, identifying a novel oncogenic role for NEAT1, that of inducing cellular autophagy through GABARAP expression, which promotes radioresistance. Hence, NEAT1 and GABARAP are attractive molecular targets that could improve radiation therapy [224].

In 2019, Shen et al. observed an increased hepatic expression of HULC lncRNA in rat models of NAFLD. Furthermore, the research group demonstrated that inhibition of HULC expression was associated with improved pathological state and liver-function-related indexes of hepatic lipid deposition, improved degree of hepatic fibrosis, reduced hepatocyte apoptosis but, also, the inhibition of MAPK signaling pathway, evidence that all supports a clear association between increased HULC expression and NAFLD progression [225]. A few years before, Li et al. reported an elevated expression of HULC in the liver tissue of 39 HCC patients compared with 21 healthy controls. This upregulation was positively correlated with their clinical stage, suggesting that HULC may also be involved in the development and progression of HCC. The increased expression of HULC was also confirmed in Huh-6, Huh-7, HepG2, BEL-7402, MHCC-97H, Sk-Hep1, and SMMC-7721 cell lines. At the same time, the knockdown of HULC resulted in an inhibition of cellular proliferation and in the induction of cellular apoptosis in vitro. Moreover, their research data indicate that HULC negatively regulates miR-200a-3p, upregulating ZEB1 expression in HCC cells, which enhances EMT. Finally, it was also observed that HULC is involved in tumor growth and intrahepatic metastasis, thus being considered a promoter of HCC pathogenesis through miR-200a-3p/ZEB1 EMT pathway [226]. Altogether, these findings highlight a promising research direction, but extensive studies are required for a complete understanding of HULC implication in NAFLD-related HCC.

Wang et al. aimed to investigate which part of SNHG20 lncRNA plays in the pathogenesis of NAFLD-related HCC and whether polarization of liver resident macrophages, Kupffer cells (KCs), is a contributive factor. Their findings showed that SNHG20 expression is decreased in human NAFLD but increased in human NAFLD-HCC livers, the results are also consistent in the mouse models. Both human and mouse NAFLD KCs displayed M1 polarization compared with NAFLD-HCC KCs, while silencing SNHG20 induced M1 polarization in RAW264.7 macrophages and delayed the progression of NAFLD to HCC in the animal model. However, SNHG20 overexpression induced M2 polarization by activating STAT6 in RAW264.7 macrophages. Based on these results, SNHG20 could be a viable therapeutic target against NAFLD progression to HCC, as it regulates liver KCs polarization [227].

Previous studies have shown that hepatic cholesterol is one of the main lipotoxic molecules involved in the progression of NAFLD to HCC [228,229,230]. In a recently published article, Liu et al. orchestrated a comprehensive study design conducted on HCC patients, human liver cell lines HepG2, HEK293T, and Huh-7 cells, cholesterol-driven C57BL/6J NAFLD–HCC mice, liver orthotopic xenograft tumors, and on patient-derived xenograft (PDX) tumors with high expression of SNHG6. The researchers observed a dynamic interplay between cholesterol and the SNHG6, a putative cholesterol effector, and their engagement in a self-amplified cycle that accelerated the NAFLD-related HCC development. From a mechanistic point of view, it was shown that cholesterol binds to the ER-anchored FAF2 protein, which allows the formation of the SNHG6–FAF2–mTOR complex, through which the SNHG6 co-ordinates the mTORC1 kinase cascade activation and cellular cholesterol biosynthesis in a self-amplified cycle that speeds up the cholesterol-driven NAFLD to HCC transition. Moreover, the loss of SNHG6 expression was responsible for mTORC1 signaling inhibition and the growth impairment of PDX liver tumors, promoting SNHG6 as a promising biomarker for developing HCC targeted therapies [231].

In 2020, Wang et al. found that GAS5 lncRNA is downregulated in tumor tissue samples collected from HCC patients and in HCC HepG2 and HepB3 cell lines. This downregulation of GAS5 expression contributed to an elevated level of miR-21 and, later, to suppression of expression of PTEN, which finally resulted in HCC cell proliferation, inhibition of cellular apoptosis, and increased resistance to doxorubicin treatment. Hence, the researchers highlight the potential of restoring GAS5 expression as a new therapeutic approach for HCC [232]. One year later, Cui and his research group investigated the same lncRNA in a NAFLD C57BL/6 mice model and revealed that GAS5 and NOTCH2 expression is elevated, while miRNA-29a-3p expression is decreased. They concluded that GAS5 overexpression augmented NOTCH2 levels in liver cells and promoted NAFLD progression by sponging miR-29a-3p in vivo. To prove these findings, the researchers also demonstrated that GAS5 knockdown attenuated hepatic steatosis and lipid accumulation, thus reducing the NAFLD activity score in HFD mice. Moreover, GAS5 knockdown caused a reduction in serum triglyceride cholesterol levels while inhibiting alanine aminotransferase and aspartate aminotransferase activities in vivo. Their results suggest that GAS5 is a potent regulator of the miR-29a-3p/NOTCH2 axis while actively involved in NAFLD progression, which recommends it as a potential therapeutic target against NAFLD [125]. These findings reflect a possible role of GAS5 in the progression of NAFLD to HCC, but extensive research and validation are required.

Wu et al. performed a series of comprehensive investigations focused directly on a cohort of NAFLD-related HCC cases and in vivo and in vitro lab-grown study models. M2 to M1 phenotype is an essential transition in cancer development that can reverse the tumor-promoting effects of M2 macrophages [227,233,234]. The expression of lncRNA FTX and M1/M2 KCs ratio decreased during the NAFLD conversion to HCC. At the same time, upregulation of FTX inhibited this pathological transition via promoting KCs polarization to M1 phenotype [233]. Chi et al. investigated the role of lncARSR in NAFLD and its role in the progression towards HCC. They revealed an elevated expression status of lncARSR in HFD-fed mice and FFA-treated HepG2 cells. Moreover, they observed that lncARSR binds to YAP1, a crucial component of the Hippo pathway and a regulator of HCC progression, thus blocking its nuclear translocation while activating IRS2/AKT pathway further to increase lipid accumulation, proliferation, and invasion. LncARSR proves to be involved in NAFLD and HCC through the regulation of the YAP1/IRS2/AKT axis, while silencing its expression seems to reduce lipid accumulation in the NAFLD mice model [235].

MALAT1 lncRNA expression was elevated in liver tissue samples of NAFLD patients, serum samples of HFD-fed mice model of NAFLD, and inHepG2 cells treated with 1 mM of FFA (in vitro model of NAFLD). The researchers demonstrated that knockdown of MALAT1 upregulated the expression of PPARα and reduced CD36 levels, thus reversing fatty-acids-induced lipid accumulation in HepG2 cells. In addition, knockdown of MALAT1 was found to be responsible for the reduced levels of miR-206, while it also increased the expression of ARNT, which is known to be involved in NAFLD development. These findings pinpoint the PPARα/CD36-mediated hepatic lipogenesis roles of MALAT1 in NAFLD through regulating the miR-206/ARNT axis [236]. Wu et al. reported that MALAT1 is an active promoter of NAFLD-associated fibrosis as it was found to upregulate post-TGF-β1 exposure and was associated with a reduced expression of SIRT1 in CCL4-treated mice and in LX-2 HSCs [237]. At the same time, Malakar et al. described MALAT1 as a critical oncogene involved in the upregulation of SRSF1 and the activation of the Wnt pathway, thus promoting HCC growth and development in liver tumors of HCC mouse model [238]. Therefore, although the current lack of data limits understanding of the implications of MALAT1 in NAFLD-related HCC pathogenesis, these findings highlight a promising research avenue.

A summary of other dysregulated lncRNAs that could play a part in the NAFLD-related HCC pathogenesis and deserve further investigation is presented in the following table (Table 2).

## 7. Conclusions

The medical advances that lead to a decrease in hepatitis B and hepatitis C infection, combined with changes in the populational diet and disease prevalence with an increase in obesity and T2D, indicate that NAFLD will become the leading risk factor associated with the development of HCC.

In this regard, the need to reconsider HCC clinical guidelines to include clear screening and clinical surveillance recommendations for patients with noncirrhotic NAFLD. Progresses in genomic medicine led to the identification of multiple regulatory pathways involved in the development of NAFLD and the progression of NAFLD towards HCC. Among the newly discovered regulatory elements, a particular interest has been decoding the roles of ncRNAs in modulating these regulatory pathways. These ncRNA molecules, represented mainly by miRNAs and lncRNAs, play a central regulatory role.

A better understanding on the progression of NAFLD towards HCC and molecular alterations present in the tumor cells and surrounding tumor microenvironment can be achieved by the study of molecular markers, such as ncRNAs, that represent an important tool for decoding cellular dynamics during early-tumor progression. Functional ncRNA species are mainly represented by miRNA, lncRNAs, and, more recently, an important regulatory role seems to be associated with circularRNAs. These ncRNAs species were shown to be important regulators of the important processes involved in HCC development and progression, such as chronic inflammation, maintaining a hypoxic microenvironment, and angiogenesis.

Chronic inflammation is an important pathogenic factor in the development of NAFLD and further in the progression towards HCC. In addition to metabolic stressors that are known to be involved in the initiation and progression of inflammation, we showed that an important regulatory role at a molecular level is represented by miRNAs and lncRNAs that orchestrate a complex network. Evaluation of the dynamics of these molecules can identify at-risk patients that can be further screened or supposed to therapeutic interventions to limit further disease progression.

Recent evidence showed that ncRNAs are involved in the regulation of hypoxia in HCC via HIF-1α and are responsible for the development of an immunosuppressive tumor microenvironment, which facilitates immune evasion of tumor cells [249]. A hypoxic environment further promotes angiogenesis, which enhances tumor growth and dissemination. Therefore, decoding the regulatory ncRNA network involved in the promotion of angiogenesis can identify new approaches of limiting tumor growth and dissemination [250].

Understanding their dynamics in NAFLD and HCC can identify new biomarkers and therapeutic targets that will allow a better future follow-up and management of these patients. Therefore, our review aims to provide an up-to-date analysis of recent advances in the field of ncRNA involvement in NAFLD development and progression, focusing on possible translational usage of these molecules.

## Figures and Tables

**Table 1:** Other dysregulated miRNAs involved in both NAFLD and HCC.

miRNA	Expression	Study Sample	Molecular Targets	Effects	Refs.
miR-23a	↑	Liver tissue samples from a murine model of NAFLD-related HCC	↓ PGC-1α ↓ G6PC	Inhibition of gluconeogenesis	[193]
miR-33	↓	NAFLD C57BL/6N mice	↑ SREBP1	Hepatic lipogenesis	[194]
	↑	Serum samples of patients with NAFLD after liver transplantation	↑ SREBP1/2	Increased steatosis. Increased inflammation	[195]
	↑	86 pairs of primary HCC tissues and adjacent nontumor liver tissues Human HCC cell lines, HepG2, Huh7	↓ PPAR-α	Induced cellular proliferation. Inhibition of apoptosis	[196]
miR-15b	↑	One NAFLD group of 8 SD rats fed with HFD and one control group of 8 SD rats fed with normal diet. Diet-induced obesity C57BL/6N mice	↓ INSR ↓ CCNE1 ↓ LPK ↑ PPRC1	Increased hepatic insulin resistance Decreased cell proliferation Decreased glucose utilization Decreased triglyceride metabolism	[197,198,199,200,201,202]
	↑	Human liver L02, QSG7701, HepG2 cell lines Serum samples from 69 NAFLD patients and 42 healthy subjects			
	↑	25 pairs of primary HCC and adjacent nontumor liver tissues Human HCC cell lines SNU475, SNU761, Huh-7, and HepG2, one cholangiocarcinoma cell line (KMBC) and immortalized human hepatocytes. Plasma samples of 37 HCC patients, 29 cirrhosis patients, 31 healthy cases	↓ Bcl-w	Negatively correlated with HCC recurrence Reduction in cell proliferation Enhancement of cellular apoptosis	[203,204]
	↑	STAM steatotic mice, NASH-fibrotic, and full-fledged HCC stages of liver carcinogenesis	↑ TGF-β ↑ Wnt/β-catenin	Hepatocarcinogenesis	[205]
miR-181	↑	25 patients with NAFLD and 25 healthy controls HFD-fed C57BL/6 mice Human HCC cell line HepG2	↓ SIRT1	Alleviation of hepatic steatosis	[206]
	↑	HFD-fed C57BL/6 mice Human HCC HepG2, Hep3B cells	↑ TGF-β ↓ TIMP3	Promotion of hepatocarcinogenesis	[207]
miR-143	↑	Human white pre-adipocytes	↑ ERK5	Adipocyte differentiation	[208]
	↑	Human HCC cell line HepG2	↑ NF-κB	Promotion of cell migration	[209]
miR-192	↓	Nonalcoholic steatohepatitis rat models fed with HFD	↑ SCD-1	Negative regulatory role in lipid synthesis	[210]
	↓	101 HCC primary tumors and adjacent nontumor tissues Human HCC cell lines Huh-7, SK-Hep-1, SNU-449, and HEK-293T, MHCC- 97L, MHCC-97H, and LM3	_	_	[211]
	↑	miR-192 mimic transfected human HCC cell lines HCC-LM3, Huh-7, and SK-Hep-1	↓ SLC39A6/SNAIL	Reduction in tumor metastasis	
miR-194	↑	Human-derived HepG2 cells HFD-fed C57BL/6J mice	↓ FXR/Nr1h4	NAFLD development Disruption of hepatic inflammatory response Metabolic disruption	[212]
	↑	Tumor tissues and adjacent healthy liver tissues from 131 HCC patients HCC cell lines SMCC7721, LM3, Hep3B, MHCC97H, and normal hepatic cell line THLE-3	↓ CADM1	Increased malignant behavior of HCC cells. Increased proliferation of HCC cells in vivo Increased growth of HCC tumors in vivo	[213]

**Table 2:** Other dysregulated lncRNAs involved in both NAFLD and HCC.

lncRNA	Expression	Study Sample	Molecular Targets	Effects	Refs.
H19	↑	HFD NAFLD C57BL/6mice model FFA-treated HepG2 and Huh-7 cells	↑ PPARγ ↑ ACC1 ↑ SCD1 ↑ FASN ↑ SREBP1 ↓ miR-130a	Increased hepatic lipogenesis NAFLD progression	[239]
	↑	46 Fresh-frozen HCC tissues and matched adjacent normal liver tissues HCC cell line HepG2, SMMC-7721, Bel-7402, Huh-7, and human normal liver cell lines WRL-68,293T	↑ CDC42/PAK1 ↓ miR-5b	Promotes EMT Promotes HCC cells proliferation, migration, and invasion HCC progression	[240]
HOTAIR	↑	HFD-induced NAFLD mice model FFA-treated HepG2 cells	↓ miR-130b-3p/ROCK1	Lipid accumulation	[241]
	↑	46 HCC tissue samples HCC cell lines L02, HepG2, Bel-7402, MHCC97L, MHCC97H, HCCLM3, HEK-293T	↑ FUT8/α-1,6 core-fucosylatedHsp90/ MUC1/STAT3 ↑ JAK1/STAT3	HCC progression	[242]
MEG3	↓	HFD-fed NAFLD C57/BL6 mice	↑ SIRT6 ↓ EZH2	Protective effects against lipogenesis and inflammation	[243]
	↓	HCC tissues matched with adjacent normal tissues from 30 patients HCC cell lines SK-HEP-1, Huh-7, and embryonic kidney cell line 293T	↑ miR-9-5p ↓ SOX11	Inhibition of HCC cells growth	[244]
RMRP	↑	Liver tissues from 30 NAFLD patients and 30 healthy volunteers FFA-treated AML-12 cells	↑ miR-206 ↑ PTPN1	NAFLD progression, but its downregulation has an opposite effect	[245]
	↓	HCC cell lines Huh7, HLE	↑ PERK	Induced apoptosis of HCC cells	[246]
HCG18	↑	Serum samples from 116 NAFLD patients and 101 healthy controls HFD-fed NAFLD C57Bl/6 mice	↑ miR-197-3p IL18	Promotes insulin resistance and fat accumulation	[247]
	↑	60 HCC tissues paired with adjacent normal ones from HCC patients - HCC cell lines Huh-7, MHCC97-H, and normal liver cell line THLE-2	↓ miR-214-3p ↑ CENPM	Promotes the proliferation and migration of HCC cells	[248]
